# Lipid‐Based Nanoparticles in Cancer Therapy: Advances in Targeted Drug Delivery and Therapeutic Potential for Renal Cell Carcinoma

**DOI:** 10.1002/advs.202507666

**Published:** 2025-08-28

**Authors:** Wei Yao, Yuhe Lin, Yang Weng, Yan Wu, Lufeng Zheng, Donghan Zheng, Qi Xi, Jingying Zhao

**Affiliations:** ^1^ Department of General Surgery Shengjing Hospital of China Medical University Shenyang Liaoning 110000 China; ^2^ Department of Oncology Shengjing Hospital of China Medical University Shenyang Liaoning 110000 China; ^3^ Department of Digestive Endoscopy The Fourth Affiliated Hospital of China Medical University Shengyang 110032 China; ^4^ School of Life Science and Technology China Pharmaceutical University 639 Longmian Road Nanjing Jiangsu Province 211198 China; ^5^ Department of Cardiology The Fourth Affiliated Hospital of China Medical University Shenyang 110032 China; ^6^ Department of Pain Medicine The First Hospital of China Medical University Shenyang 110001 China; ^7^ Department of Nephrology Shengjing Hospital of China Medical University Shenyang 110004 China; ^8^ Department of Pediatrics Shengjing Hospital of China Medical University Shenyang 110004 China

**Keywords:** drug resistance, enhanced permeation and retention effect, lipid nanoparticles, renal cell carcinoma, targeted drug delivery

## Abstract

Renal cell carcinoma (RCC) remains a significant clinical challenge due to delayed diagnosis and multidrug resistance, necessitating innovative strategies to enhance early detection and therapeutic efficacy. This review evaluates the role of lipid‐based nanoparticles (LNPs), including liposomes, micelles, nanoemulsions, solid lipid nanoparticles, and nanostructured lipid carriers, as advanced drug delivery systems in providing innovative solutions for RCC diagnosis and treatment. These LNPs exhibit unique advantages of great biocompatibility, biodegradability, extended circulation, and tumor‐targeting capabilities, which enable precise drug delivery, controlled release, and circumvention of drug resistance mechanisms. After integrating recent advancements, it is highlighted that formulations such as Liposome@Sunitinib can enhance tumor accumulation, two‐drug‐loaded liposomes can activate immune responses, and RNA therapy in combination with immunomodulators can reverse drug resistance. Current findings underscore the transformative potential of lipid‐based nanotechnology in addressing RCC‐specific challenges, particularly in overcoming biological barriers and refining personalized treatment approaches. In the future, multifunctional nano platforms, precision therapies based on patient genes, advanced materials engineering, combination therapies, and sustainable manufacturing can be developed to promote the application of LNPs in the treatment of RCC and realize personalized nanomedicine.

## Graphical abstract

This review explores the transformative potential of lipid‐based nanoparticles (LNPs) in renal cell carcinoma (RCC) therapy. It systematically analyzes six LNP platforms‐from liposomes to hybrid nanoparticles‐highlighting their unique advantages in targeted drug delivery, resistance reversal, and immunomodulation. The article evaluates cutting‐edge formulations, delivery mechanisms, and clinical prospects while addressing current challenges in personalized nanomedicine for RCC.

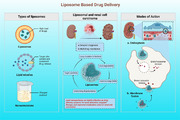

## 1 Introduction

Globally, cancer still remains a significant health challenge, accounting for ≈10 million deaths.^[^ ^1^ ^]^ Particularly in the United States alone, cancer is the second leading cause of death after heart disease.^[^ ^2^ ^]^ In 2024, it is projected that over 2 million new cancer cases will be diagnosed, with ≈611720 expected deaths.^[^ ^2^ ^]^ Among the potential cancers of unknown primary site (CUP), most of them belong to unfavorable subsets, with a modest sensitivity to therapy and a median overall survival (OS) of generally 6–10 months.^[^ ^3^ ^]^ Different subtypes of renal cell carcinoma (RCC) originate in the renal epithelial tissue, and among these, 2% of the cases root from inherited syndromes.^[^ ^4^, ^5^ ^]^ Histological and molecular subtypes identify clear cell carcinoma as the most common form of RCC, frequently caused by mutations in the VHL gene, resulting in the aberrant accumulation of hypoxia‐inducible factors (HIFs) and the activation of pro‐angiogenic signaling cascades, notably the vascular endothelial growth factor (VEGF) pathway.^[^ ^6^ ^]^ Although surgical therapy is the primary treatment for RCC with a favorable overall prognosis,^[^ ^7^ ^]^ recent medical advancements of targeted therapeutics, including multi‐targeted tyrosine kinase inhibitors (TKIs) and mTOR inhibitors, have introduced multiple therapeutic options for advanced or metastatic RCC. Targeted regimens mainly focus on critical signaling molecules and cascades driving RCC proliferation, such as the VEGF pathway, which promotes tumor angiogenesis, and the mTOR pathway, which governs pivotal cellular functions. Tyrosine‐kinase inhibitors (TKI) such as sorafenib and axitinib are typically administered after first‐line immunotherapy combination therapy for RCC, serving as great second‐line agents in progression‐free survival (PFS) benefits.^[^ ^8^ ^]^ mTOR inhibitors, including everolimus and temsirolimus, were approved for the treatment of advanced RCC.^[^ ^9^ ^]^ These newer agents have largely replaced conventional cytokine‐based therapies, which were associated with moderate response rates and significant side effects.^[^ ^10^ ^]^ The goal is to transition from a “one‐size‐fits‐all” approach to a more personalized and targeted treatment.^[^ ^11^, ^12^ ^]^ RCC patients who have developed distant metastasis, or when the tumor is too large and the local anatomy of the tumor growth site is complex, RCC exhibits resistance to radiotherapy and chemotherapy. In such cases, clinical practice primarily relies on targeted drugs as a supplementary treatment.^[^ ^13^ ^]^ Recently, the continuous advancements in medical diagnostic technology have contributed to heightened rates of early RCC detection and favorable clinical prognoses. Importantly, exosomes have emerged as a novel source of non‐invasive tumor biomarkers, especially RCC.^[^ ^14^ ^]^ The unique bilayer membrane structure of exosomes offers protection against external RNases and proteases, leading to enhanced stability of the enclosed mRNAs, miRNAs, and functional proteins, thus making exosomes highly sensitive markers for disease diagnosis. The cargo in tumor‐derived exosomes, such as the range of miRNAs, can also serve as biomarkers for RCC in the serum and urine of patients, offering valuable targets for early detection and monitoring of the disease.^[^ ^15^ ^]^ Such advancements are critical, early prevention and treatment remain a significant challenge in RCC patients.^[^ ^16^ ^]^

In recent years, nanoparticles have emerged as a promising approach in cancer therapy, offering improved efficacy and unique advantages over traditional treatments.^[^ ^17^ ^]^ Nanoparticles, particularly lipid‐based nanoparticles, have garnered significant attention due to their unique properties, which enable targeted drug delivery, enhanced cytotoxicity, and reduced adverse effects. These nanoparticles, which carry pharmaceutically active agents, typically range in size from 1 to over 500 nm and demonstrate improved bioavailability of the encapsulated drug(s). Among the various types of nanocarriers, metallic, polymeric, inorganic, organic, and lipid‐based nanoparticles are the most widely studied. The researchers have employed these nanocarriers to effectively treat cancers with improved pharmacokinetics and enhanced efficiency of the loaded approved and investigational cytotoxic drugs.^[^ ^18^, ^19^, ^20^ ^]^ Similar to the other types of nanoscaled drug delivery systems, lipid nanoparticles can incorporate, encapsulate or entrap active pharmaceutical ingredients, including drugs, peptides, nucleic acids, and other bioactive molecules.^[^ ^21^ ^]^ Among the above‐mentioned nanoformulations, the lipid nanoparticles have been reported and discussed for the added benefits toward cancer therapeutics.^[^ ^18^, ^22^, ^23^ ^]^ These added benefits include biocompatibility, biodegradability, and passive targeting with longer circulation time.^[^ ^24^, ^25^, ^26^ ^]^ These advancements have shown potential in not only accurate and early diagnosis, but also treating various cancers, including renal cancer, by improving the bioavailability and stability of anticancer drugs.^[^ ^16^, ^27^, ^28^ ^]^ The use of lipid nanoparticles is particularly advantageous due to their biocompatibility, ability to encapsulate a wide range of therapeutic agents, and potential to bypass biological barriers. This paper thoroughly reviews such innovative approaches, regarding the use of nanoparticles, holding great promise in addressing the ongoing challenges in RCC treatment, potentially leading to better patient outcomes and reduced side effects.

## 2 Renal Cancer: Cellular Mechanism of Disease Progression

**Figure 1:**
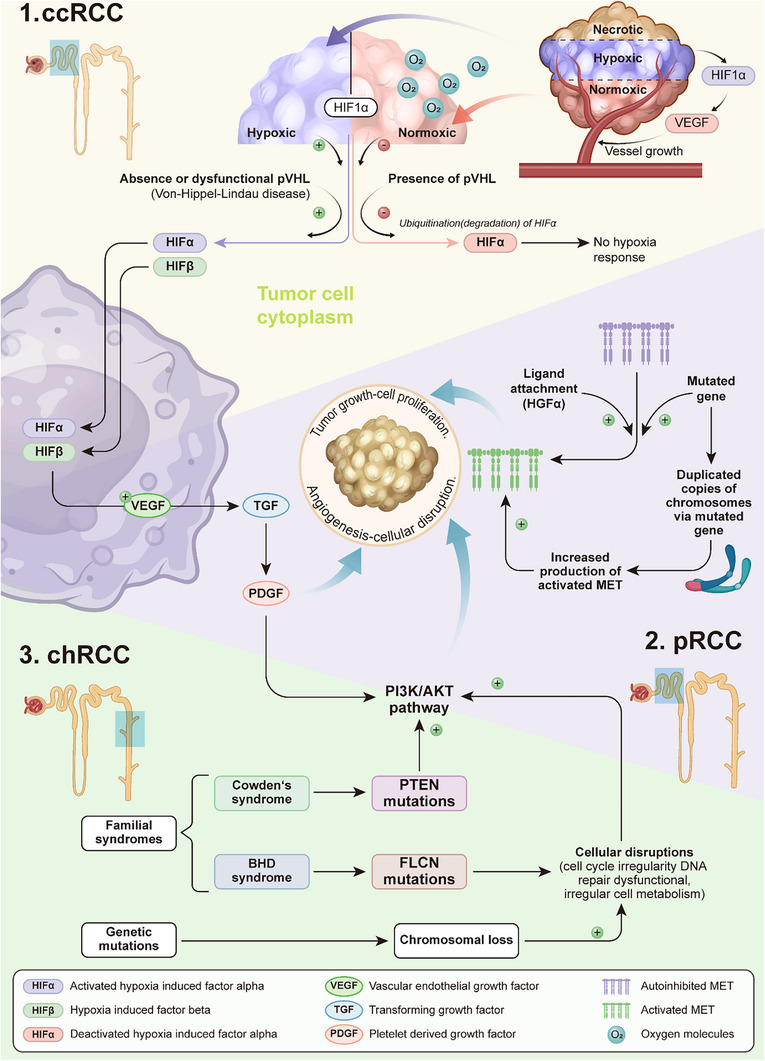
Pathophysiology of common subtypes of RCC and their associated molecular pathways. Abbreviations: PI3k/AKT: Phosphoinositide 3‐kinase/Protein Kinase B, HGF: Hepatocyte Growth Factor, MET: Mesenchymal Epithelial Transition, BHD: Birt‐Hogg‐Dubé Syndrome, PTEN: Phosphatase and Tensin Homolog, FLCN: Folliculin, ccRCC: Clear Cell Renal Cell Carcinoma, pRCC: Papillary Renal Cell Carcinoma, chRCC: Chromophobe Renal Cell Carcinoma, pVHL: Von‐Hippel‐Lindau protein.

RCC is classified into different types of diseases, such as clear‐cell subtype (ccRCC), papillary (pRCC), chromophobe (chRCC), and some miscellaneous tumors, which mimic RCC^[^ ^4^, ^29^, ^30^ ^]^ (**Figure** **1**). The pathology associated with each subtype of RCC is distinct, leading Jonasch et al. to advocate for treating these subtypes as independent entities rather than categorizing them collectively under a single umbrella term such as “subtypes.”^[^ ^29^ ^]^ RCC, particularly the clear‐cell subtype, which represents around 70% of all RCC and papillary RCC, mostly begins in the lining of the proximal renal tubules, while the chromophobe RCC is a manifestation of intercalated cells in the collecting duct.^[^ ^31^, ^32^ ^]^ The proximal renal tubule, as the initial segment of the extended renal tubule, is the first to encounter glomerular filtrates. Consequently, factors such as high cellular turnover, exposure to elevated levels of toxins and metabolites, genetic predispositions, and an ideal microenvironment contribute to the development of carcinoma in the proximal convoluted tubule.

Multiple pathways implicated in the pathogenesis of RCC have been identified in the literature. These pathways are complex and involve a series of genetic mutations and epigenetic alterations, which collectively drive uncontrolled cell proliferation, invasion of surrounding tissues, and eventual metastasis to distant organs. RCC is frequently underdiagnosed in its early stages due to the lack of apparent symptoms. By the time the most commonly reported symptoms—such as hematuria and/or flank pain—manifest, the disease has often progressed to an advanced stage.^[^ ^33^ ^]^

Von Hippel‐Landau (VHL) tumor suppressor gene holds a pivotal role in the overall pathophysiology of RCC, which is expressed by 3p25 genetic locus.^[^ ^34^ ^]^ Inactivation or absence of VHL leads to increased production of growth factors, such as vascular endothelial growth factor (VEGF), transforming growth factor (TGF), platelet‐derived growth factor (PDGF), and others. All these factors are normally produced in response to hypoxia‐induced accumulation of hypoxia inducible factor α (HIF‐α) and are responsible for tumor cell proliferation, survival, and tumor angiogenesis by acting on the neighboring vascular cells.^[^ ^35^ ^]^ In physiological states, the VHL‐HIF‐α pathway maintains oxygen homeostasis in cells and tissues through oxygen‐dependent dynamic regulatory mechanisms. VHL initiates its degradation by directly binding hydroxylated HIF‐1α.^[^ ^36^ ^]^ HIF‐1α dominates angiogenesis in the early stages of acute hypoxia, while HIF‐2α drives vascular remodeling and metabolic adaptation during persistent hypoxia.^[^ ^37^ ^]^ Indeed, the loss of functional VHL protein is considered a defining event in the development of clear‐cell RCC, and the principal downstream oncogenic mechanism appears to be HIF‐2α accumulation and constitutive HIF transcription factor activity.^[^ ^38^ ^]^ Belzutifan is a potent small‐molecule inhibitor of HIF‐2α that prevents heterodimerization with HIF‐1β into an active transcription factor and has shown activity in clear‐cell RCC. A recently published phase III study showed a significant benefit of belzutifan over everolimus concerning progression‐free survival and objective response in participants with advanced clear‐cell RCC who had previously received immune checkpoint and antiangiogenic therapies.^[^ ^39^ ^]^ Apart from its genetic drivers, RCC exhibits a highly vascularized and immunosuppressive tumor microenvironment. The overexpression of VEGF and PDGF due to VHL mutations enhances angiogenesis, while the hypoxic niche fosters metabolic reprogramming toward glycolysis.^[^ ^40^, ^41^, ^42^ ^]^ This hypoxic and immunosuppressive microenvironment poses a significant challenge for conventional chemotherapy and immunotherapy, necessitating innovative drug delivery systems to overcome these barriers.

In papillary renal cell carcinoma (pRCC), dysregulation of the Mesenchymal‐Epithelial Transition factor (MET) pathway has been identified as a primary driver of tumorigenesis. MET dysregulation is a key driver in type 1 pRCC, which benefits from MET‐targeted therapy, whereas it has a limited role in type 2 pRCC with a lower MET mutation rate. Tumor progression of type 2 pRCC is more dependent on fumarate accumulation and HIF‐1α/2α activation due to FH deletion.^[^ ^43^ ^]^ MET, a receptor tyrosine kinase, is typically inactive in the absence of its ligand, hepatocyte growth factor (HGF). However, in pRCC and certain sporadic cases, mutations in the MET gene result in ligand‐independent activation of the receptor. Furthermore, gene duplication events leading to an increased copy number of the MET gene can amplify MET protein activation, further exacerbating oncogenic signaling and cancer progression.^[^ ^29^, ^35^ ^]^

Lastly, chRCC is the type in which losses of whole chromosomes such as 1, 2, 6, 10, 13, 17, and 21 have been reported.^[^ ^29^ ^]^ Most tumors in chRCC are sporadic, and familial forms include Birt‐Hogg‐Dubé (BHD) syndrome, which is linked to mutations in folliculin gene (FLCN). Another chRCC syndrome known as Cowden's syndrome includes mutations in the Phosphatase and Tensin Homolog (PTEN) gene, a tumor suppressor gene involved in the inhibition of the PI3K/AKT signaling pathway. These molecular mechanisms highlight how disruptions in normal cellular processes can lead to the development and progression of RCC.^[^ ^29^, ^35^ ^]^

**Figure 2:**
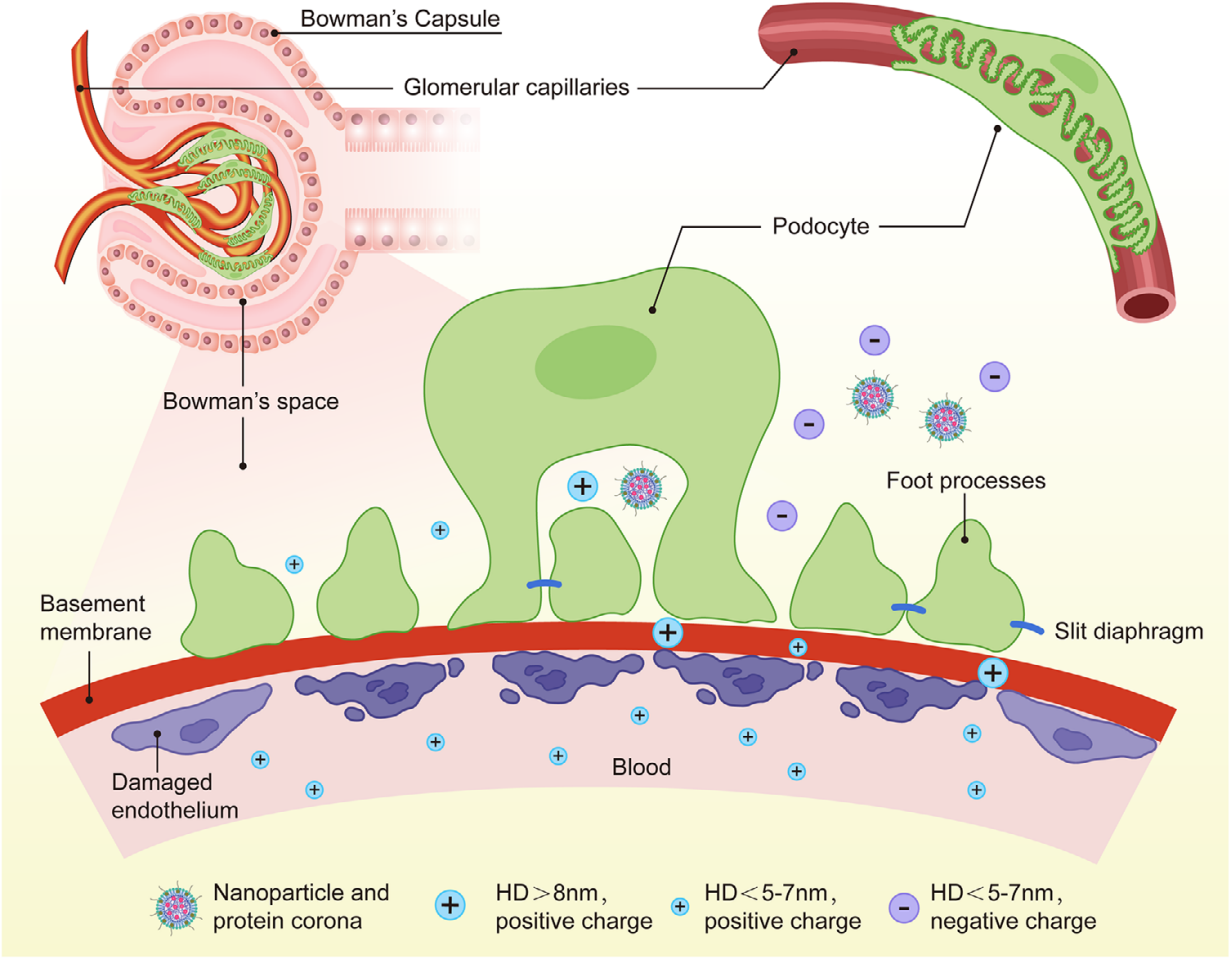
Nanoparticles passing accessing deeper renal tissue by passing through glomerulus barrier. *HD: Hydrodynamic diameter*.

Among the numerous challenges to achieving successful therapeutic outcomes in RCC, multidrug resistance (MDR) remains the most significant obstacle. However, the use of nanoscaled drug delivery systems has shown promise in overcoming this challenge.^[^ ^44^, ^45^, ^46^, ^47^ ^]^ Additionally, accessing deeper regions of the renal system and achieving targeted drug delivery specifically to RCC tissues—while sparing healthy tissues—have proven difficult with conventional therapies.^[^ ^48^ ^]^ In addition to its intrinsic drug resistance, RCC frequently develops acquired resistance to tyrosine kinase inhibitors (TKIs) through VEGF‐independent angiogenesis and the upregulation of alternative pro‐survival pathways, such as MET and fibroblast growth factor receptor (FGFR) signaling.^[^ ^49^, ^50^ ^]^ Furthermore, ATP‐binding cassette (ABC) transporters, particularly ABCG2 and ABCB1, contribute to multidrug resistance by actively effluxing TKIs from cancer cells.^[^ ^51^ ^]^ Down‐regulation of lactotransferrin, a significant protein in the innate immune system, promotes metastasis in RCC. Interestingly, this down‐regulation also renders the RCC tumor cells more responsive to mTOR inhibitors, suggesting its potential as a predictor for therapeutic effectiveness.^[^ ^52^ ^]^ Immunotherapy resistance in RCC is also influenced by an immunosuppressive microenvironment, characterized by increased infiltration of regulatory T cells (Tregs) and myeloid‐derived suppressor cells (MDSCs), as well as adaptive resistance mechanisms like upregulated autophagy pathways.^[^ ^53^, ^54^, ^55^, ^56^ ^]^ To address these limitations, nanomedicine has emerged as a promising strategy, enabling the effective and efficient delivery of both existing and investigational drugs. This approach has the potential to enhance the eradication of deeply situated tumors. **Figure** **2** illustrates the ability of nanoscaled carriers to penetrate renal tissues.

## 3 Lipid Nanoparticles (LNPs)

**Figure 3:**
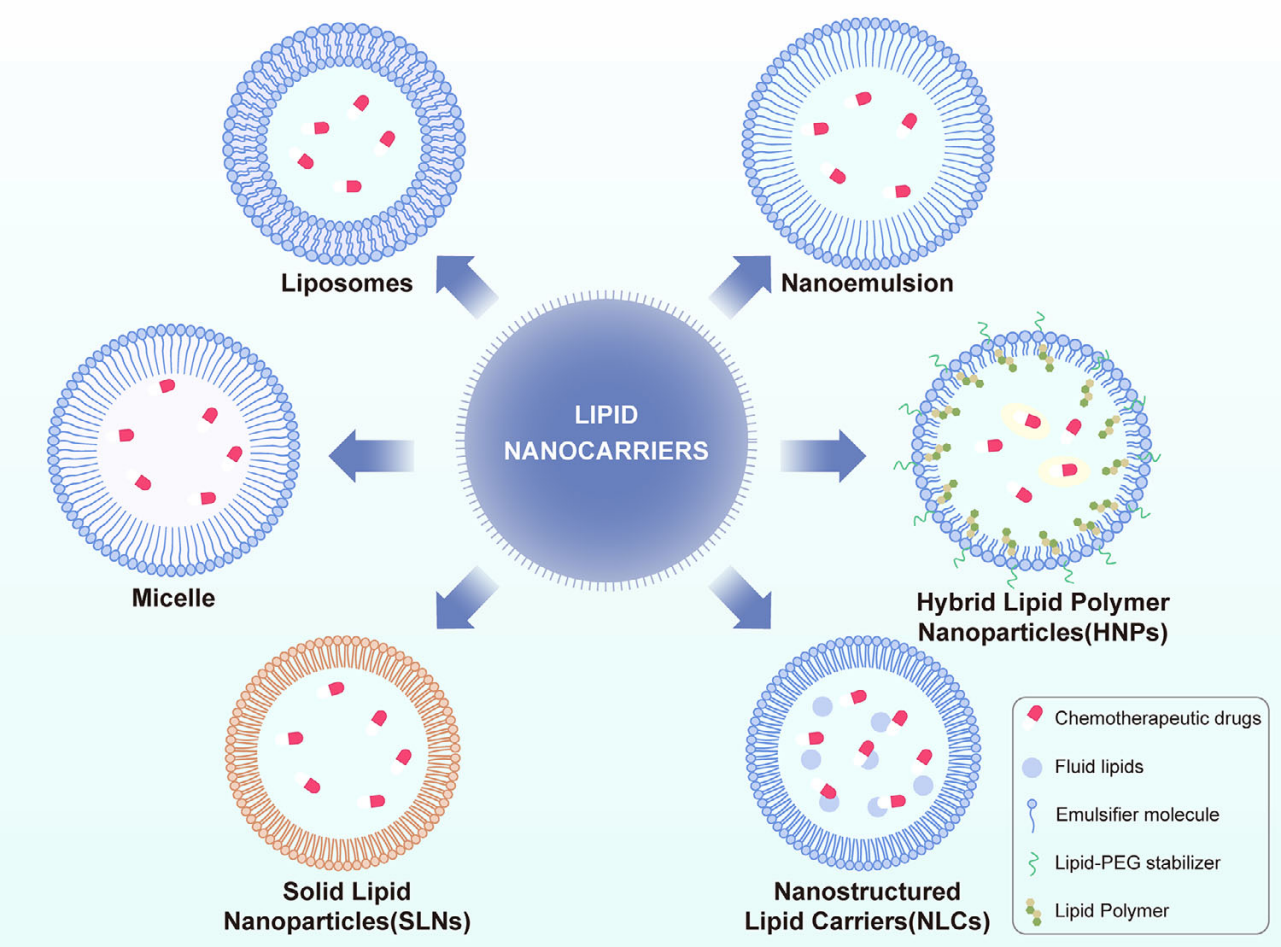
Types of Lipid‐based Nanocarriers.

In biomedical drug delivery, lipid‐based nanoparticles—such as liposomes, lipid micelles, lipid‐based nanoemulsions, solid lipid nanoparticles, nanostructured lipid carriers, and lipid‐polymer hybrid nanoparticles—are widely utilized due to their unique properties, advantages, and therapeutic potential across a variety of cancer types.^[^ ^24^, ^26^ ^]^ One of the unique advantages of lipid nanoparticles is their ability to enhance the solubility of poorly water‐soluble drugs, thereby improving bioavailability. Additionally, lipid nanoparticles enable precise control over the release of active pharmaceutical ingredients. These systems are widely favored as drug delivery platforms due to their high drug encapsulation efficiency, superior scalability, and reduced toxicity profiles. Furthermore, stimuli‐responsive lipid nanoparticles, such as pH‐sensitive formulations, can be engineered to release drugs selectively in acidic or basic microenvironments. Lipid nanoparticles can also be functionalized with active ligands that recognize and target specific tumor tissues via receptor‐mediated interactions.^[^ ^57^ ^]^ Numerous types of lipid‐based nanoparticles have been developed for anticancer drug delivery, some of which are discussed below (**Figure** **3**).

### 3.1 Liposomes

Liposomes are amphiphilic vesicular drug delivery systems typically composed of phospholipid bilayer (s) that can encapsulate both hydrophilic and/or lipophilic drugs of choice.^[^ ^58^ ^]^ They are one of the most studied drug delivery systems owing to their enhanced biodegradability and biocompatibility properties. Cholesterol is another constituent of the liposomes when added can further increase the stability of the lipid bilayers, and the permeability of poorly water‐soluble drugs through the phospholipid bilayer membrane is also increased.^[^ ^59^ ^]^ The liposomes are classified with respect to their size and number of bilayers. The multilamellar vesicles (MLVs) are usually larger and exceed 500 nm, depending upon the number of bilayers. Likewise, the liposomal vesicles of single bilayer are called giant, large, medium or small unilamellar vesicles based upon their sizes ranging from 10 to 300 nm even more. In recent years, liposomes have been studied extensively for the delivery of nucleic acids, small drug molecules, peptides, and proteins. Liposomes can be easily functionalized for targeted drug delivery with sustained release kinetics and enhanced therapeutic index of drugs.^[^ ^60^ ^]^ Currently, the commonly used functionalized liposomes include ligand‐functionalized liposomes, biomimetic cell membrane‐coated nanocarriers, PEGylated liposomes, and stimuli‐responsive liposomes.

In recent years, liposomes have been extensively investigated for cancer therapy owing to their exceptional properties as drug carriers. Numerous clinical trials are currently underway to evaluate the efficacy of liposomal delivery systems for anticancer agents.^[^ ^61^ ^]^ The synergy between resveratrol liposomes and sorafenib boosts anti‐RCC tumor efficacy and helps counteract sorafenib resistance in RCC models through modulation of the PI3K‐AKT‐mTOR and VHL‐HIF pathways.^[^ ^62^ ^]^ Previous study engineered liposomes containing triptolide (TP) and targeted with Asn‐Gly‐Arg (NGR) peptide conjugated mPEG2000‐DSPE to induce normalization of tumor blood vessels, thereby boosting the sensitivity of tumor cells to radiotherapy.^[^ ^63^ ^]^ Carnosic acid (CA) was encapsulated in liposomes, which were linked to transferrin to effectively address issues of low solubility and absorption at the site of the lesion.^[^ ^64^ ^]^

**Figure 4:**
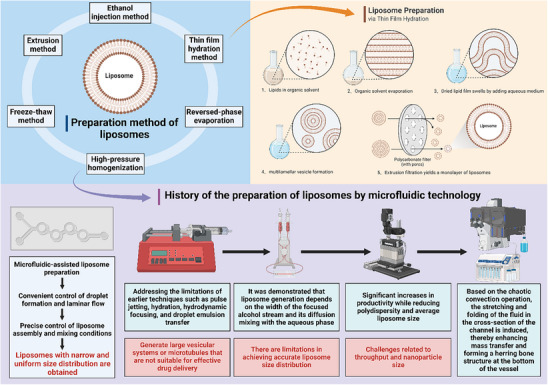
Evolution of microfluidic‐assisted liposome synthesis technology: From traditional methods to precision nanoparticle fabrication.

Traditional methods for preparing liposomes include sonication,^[^ ^65^ ^]^ thin film hydration,^[^ ^65^, ^66^ ^]^ extrusion,^[^ ^67^ ^]^ ethanol injection,^[^ ^68^ ^]^ high pressure homogenization,^[^ ^69^, ^70^ ^]^ reverse‐phase evaporation,^[^ ^71^ ^]^ and freeze thawing.^[^ ^72^ ^]^ Among these, freeze‐thawing, extrusion, ultrasonication, and high‐pressure homogenization can be combined with more modern techniques. One such advanced method is microfluidic‐assisted liposome formulation, which leverages the precise manipulation of fluids in microchannels with dimensions ranging from tens to hundreds of micrometers.^[^ ^73^, ^74^ ^]^ Microfluidics offers unique advantages, such as droplet formation and laminar flow, enabling precise control over liposome assembly and mixing conditions, resulting in liposomes with narrow and uniform size distributions. The microfluidic hydrodynamic focusing method, first reported by Jahn et al., addressed limitations of earlier techniques like pulsed jets, hydration, hydrodynamic focusing, and droplet emulsion transfer, which often produced large vesicular systems or microtubules unsuitable for effective drug delivery.^[^ ^75^ ^]^ In this method, lipids spontaneously self‐assemble into liposomes when aqueous solutions from two side channels mix with a central channel containing lipids dissolved in an organic solvent.^[^ ^76^ ^]^ Jahn et al. further refined the system to improve liposome uniformity and size, demonstrating that liposome generation depends on the width of the focused alcohol stream and its diffusion mixing with the aqueous phase.^[^ ^77^ ^]^ While traditional 2D hydrodynamic focusing using chip‐type devices offers some control, it has limitations in achieving precise liposomal size distributions.^[^ ^78^ ^]^ To overcome this, Hood et al. developed a 3D microfluidic hydrodynamic focusing technique using concentric capillary arrays, significantly enhancing production efficiency while reducing polydispersity and mean liposome size.^[^ ^79^ ^]^ Koh et al. later introduced a five‐inlet system for microfluidic hydrodynamic focusing, producing oligonucleotide‐loaded liposomes with even smaller sizes, greater uniformity, and narrower size distributions.^[^ ^80^ ^]^ Hood et al. also designed a device with a high depth‐to‐width aspect ratio (100:1) for vertical liquid flow, enabling the production of small unilamellar and monodispersed liposomal vesicles at high production rates.^[^ ^81^ ^]^ Despite these advancements, microfluidic hydrodynamic focusing techniques faced challenges related to throughput and nanoparticle size. Stroock et al. addressed these issues by developing a staggered herringbone mixer, which produces liposomes with smaller and more uniform sizes.^[^ ^82^ ^]^ A commercial version of this device, introduced by Zhigaltsev et al., operates on chaotic advection, inducing stretching and folding of fluids across the channel cross‐section, thereby enhancing mass transfer and creating a herringbone structure at the vessel's base.^[^ ^83^, ^84^ ^]^ Maeki et al. demonstrated that increasing the flow rate ratio of fluids simultaneously reduces liposome polydispersity and size^[^ ^85^, ^86^, ^87^ ^]^ (**Figure** **4**). However, these micromixers are associated with higher costs and lower production speeds. To address this limitation, toroidal mixers have been proposed as an alternative, generating centrifugal forces and vortices that enhance chaotic advection, thereby improving mixing efficiency and throughput in liposome preparation.^[^ ^88^ ^]^

### 3.2 Solid Lipid Nanoparticles

Solid lipid nanoparticles (SLNs) represent a well‐established class of colloidal drug delivery systems, composed of various lipids to form nanoparticles ranging from 50 to 1000 nm in size. These nanoparticles remain solid at both room and body temperatures. Their matrix typically consists of mono‐, di‐, or triglycerides, glyceride mixtures, and fatty acids, stabilized by surfactants and/or polymers. SLNs have been extensively investigated as drug delivery systems due to their advantages, including enhanced physical stability, targeted drug delivery, controlled release of both hydrophilic and lipophilic drugs, biocompatibility, and cost‐effective preparation methods.^[^ ^89^ ^]^ However, SLNs also exhibit certain limitations, such as relatively lower drug loading capacity and the potential for burst release of encapsulated drugs during storage and in vivo conditions.^[^ ^90^ ^]^

The unique properties of solid lipid nanoparticles (SLNs) enable the encapsulation of chemotherapeutic agents within their matrix, thereby enhancing oral bioavailability, reducing the degradation of labile anticancer drugs, and minimizing side effects through targeted drug delivery and dose reduction. Encapsulation in SLNs also improves cellular uptake, leading to enhanced anticancer efficacy. Recent advancements in SLN‐based anticancer drug delivery include the development of Niclosamide‐loaded SLNs, which improve cellular uptake and demonstrate efficacy against triple‐negative breast cancer (TNBC).^[^ ^91^ ^]^ In 2018, Eskiler et al. developed Talazoparib‐loaded SLNs to enhance the therapeutic index against TNBC, overcoming drug resistance and reducing toxicity.^[^ ^92^ ^]^ Zielińska et al. proposed another application of SLNs for the anti‐inflammatory activity of geraniol‐ and citral‐loaded nanoparticles.^[^ ^93^ ^]^ Additionally, hydrophobic anticancer agents such as Floxuridine and Indirubin have been encapsulated in SLNs to enhance cellular uptake and improve anticancer effects against various human cancer cell lines.^[^ ^94^, ^95^ ^]^ Experimental studies on these hydrophobic agents have demonstrated significant improvements in their therapeutic efficacy.

### 3.3 Nanostructured Lipid Carriers

Nanostructured lipid carriers (NLCs) are recognized as the next generation of SLNs. They consist of an unstructured matrix composed of both solid and liquid lipids, stabilized by single or mixed surfactants at concentrations typically ranging from 1.5% to 5% (w/v). The ratio of solid to liquid lipids varies significantly depending on the formulation of the NLCs, though the quantity of liquid lipid is generally lower than that of solid lipid.^[^ ^96^ ^]^ The incorporation of oils into solid lipids creates imperfections in the crystalline structure of the solid lipid matrix, enhancing the stability and drug‐loading capacity of NLCs compared to SLNs. Additionally, NLCs exhibit a lower likelihood of drug expulsion during storage.^[^ ^97^ ^]^ NLCs can be classified based on the structure of their lipid matrix, which determines drug incorporation and depends on the proportion of liquid to solid lipids. The first type, the imperfect matrix, is composed of spatially dissimilar lipids, such as glycerides derived from fatty acids. Mixing glycerides with varying carbon chain lengths and saturation levels disrupts the crystal lattice, further increasing drug‐loading capacity.^[^ ^98^, ^99^, ^100^ ^]^ The second type, the multiple‐type matrix, consists of a solid lipid matrix dispersed with numerous nanosized oil compartments. These compartments enhance drug solubility and are used to achieve delayed drug release, as the drug must diffuse through the surrounding solid lipid matrix.^[^ ^98^, ^101^ ^]^ The third type, the amorphous matrix, is formed by blending solid lipids with specific liquid lipids, such as medium‐chain triglycerides (e.g., miglyol 812) or isopropyl myristate, to create a non‐crystalline structure. This amorphous matrix prevents lipid crystallization and accommodates drugs within its structure. While hydrophilic drugs can only be incorporated in low percentages, lipophilic drugs are effectively loaded by matching their properties with the lipids used in the formulation process.^[^ ^98^, ^102^ ^]^

Newly developed nanostructured lipid‐dextran sulfate hybrid carriers (NLDCs) were successfully designed for the prolonged delivery of water‐soluble cationic mitoxantrone hydrochloride (MTO) and held promise to address multidrug resistance in renal cancer.^[^ ^103^ ^]^ Research indicated that the sustained‐release property of NLCs can prolong the half‐life of drugs and reduce the frequency of administration. For instance, NLCs loaded with doxorubicin not only enhanced the tumor‐suppressing effect but also reduced cardiac toxicity in a mouse model of renal cancer.^[^ ^104^ ^]^ Moreover, NLCs can co‐load immunomodulators (such as IL‐12 and PD‐L1 inhibitors) to synergistically activate anti‐tumor immune responses. Their lipid matrix can escape lysosomal degradation through the mechanism of membrane fusion and improve the efficiency of drug release within cells.^[^ ^105^ ^]^ Currently, several clinical trials are exploring the synergistic effect of NLCs combined with anti‐angiogenic drugs (such as sunitinib), and it is expected that this will break through the bottleneck of renal cancer resistance and promote the development of personalized and precise treatment in the future.

The preparation methods for SLNs and NLCs include high‐pressure homogenization (performed at elevated or low temperatures),^[^ ^106^, ^107^ ^]^ microemulsion,^[^ ^108^ ^]^ solvent emulsification diffusion,^[^ ^109^ ^]^ solvent emulsification evaporation,^[^ ^110^ ^]^ solvent injection,^[^ ^99^ ^]^ double emulsion technique,^[^ ^111^ ^]^ ultrasonication,^[^ ^112^ ^]^ melting dispersion method^[^ ^113^ ^]^ and nanotemplate engineering technique.^[^ ^114^ ^]^ Briefly, high‐pressure homogenization is a mechanical process that applies high shear forces to lipids by forcing them through a narrow opening under high pressure. In this method, the drug is dispersed into melted lipids within an aqueous surfactant solution at elevated temperatures. The mixture is continuously stirred using a high‐shear device to form a pre‐emulsion, which is then homogenized using a piston‐gap homogenizer to produce nanoemulsions. These nanoemulsions are subsequently cooled to room temperature, allowing the lipids to crystallize and form nanoparticles.^[^ ^115^ ^]^ Similarly, in cold homogenization, the drug is mixed with melted lipids, followed by rapid cooling using liquid nitrogen or dry ice to ensure homogeneous drug distribution within the lipid matrix.^[^ ^116^ ^]^

### 3.4 Hybrid Nanoparticles

Hybrid nanoparticles (HNPs) represent an advanced generation of nanodrug delivery systems that synergistically combine the advantages of both lipid‐based and polymeric nanoparticles.^[^ ^117^, ^118^ ^]^ These sophisticated systems feature a unique architecture where the therapeutic agent is encapsulated within a polymeric core, which is subsequently enveloped by a functional lipid shell. The surface characteristics of the lipid shell can be precisely engineered according to the specific drug requirements through the incorporation of polyethylene glycol (PEG) and various charged or zwitterionic lipids, thereby enhancing the systemic circulation time of these nanoparticles.^[^ ^119^, ^120^ ^]^ The fabrication of HNPs typically employs biocompatible polymers such as polylactic acid (PLA), polycaprolactone (PCL), poly(lactic‐co‐glycolic acid) (PLGA), poly(β‐amino ester) (PbAE), and chitosan.^[^ ^121^, ^122^ ^]^ This hybrid system integrates the benefits of polymeric nanocarriers (including polymeric micelles, nanoparticles, and polymer‐drug conjugates) with those of lipid‐based carriers (such as SLNs, NLCs, and liposomes).^[^ ^123^ ^]^ While lipid‐based nanocarriers offer notable advantages, including cost‐effective manufacturing processes and high drug encapsulation efficiency, they are associated with certain limitations, such as restricted chemical modification potential, high polydispersity indices, rapid drug release profiles, and stability concerns.^[^ ^124^ ^]^ HNPs have been specifically designed to address these limitations, offering enhanced drug loading capacity, improved biocompatibility, tunable surface properties, and customizable drug release kinetics, thereby representing a significant advancement in nanomedicine.^[^ ^117^ ^]^

**Figure 5:**
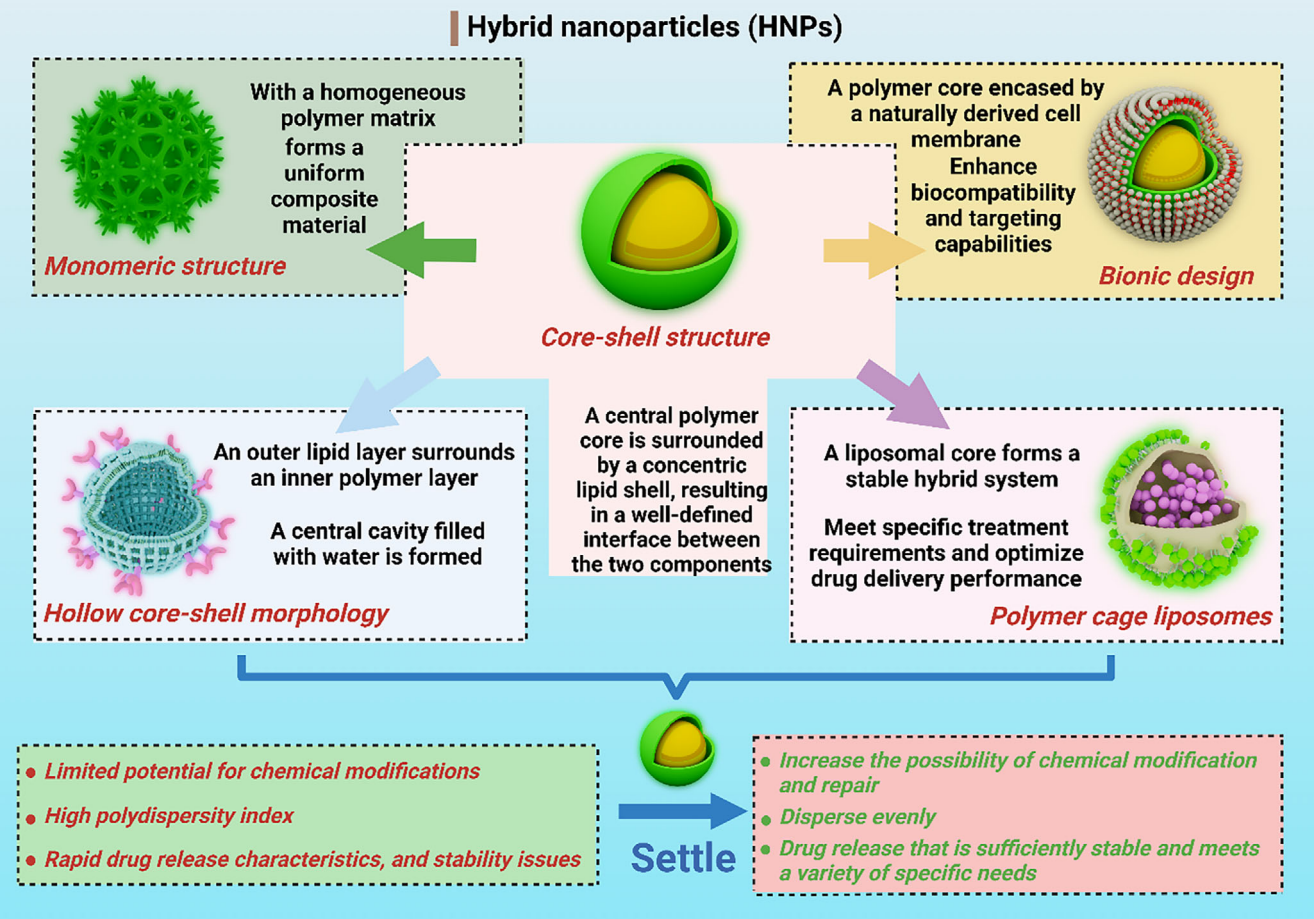
Classification of hybrid nanoparticles (HNPs) for drug delivery applications.

HNPs can be systematically categorized into five distinct structural configurations, each offering unique characteristics for drug delivery applications: 1) Core–shell architecture: This configuration features a central polymeric core enveloped by a concentric lipid shell, creating a well‐defined interface between the two components.^[^ ^125^ ^]^ 2) Monolithic structure: Characterized by a homogeneous polymeric matrix with lipid molecules randomly distributed throughout the system, forming a unified composite material. 3) Biomimetic design: Comprising a polymeric core coated with naturally derived cell membranes, this type mimics biological structures for enhanced biocompatibility and targeting capabilities. 4) Hollow core–shell morphology: Consisting of an outer lipid layer (composed of neutral or pegylated lipids) surrounding an inner polymeric layer that may interact with cationic lipids, resulting in the formation of an aqueous‐filled central cavity. 5) Polymer‐caged liposome: This unique structure involves a liposomal core with a polymer network anchored to its surface, creating a stabilized hybrid system (**Figure** **5**). These diverse architectures enable researchers to precisely engineer HNP properties to meet specific therapeutic requirements and optimize drug delivery performance.^[^ ^120^, ^126^, ^127^, ^128^ ^]^ The preparation methods for HNPs are broadly classified into two main approaches: two‐step and one‐step methodologies. The two‐step approach involves initial preparation of nanoparticles followed by their integration with liposomal components. This integration can be achieved through either electrostatic interactions between pre‐formed nanoparticles and liposomes^[^ ^129^ ^]^ or by combining polymeric nanoparticles with a dried lipid film. Both variations typically require energy input through techniques such as sonication, vortexing, or thermal treatment above the lipid phase transition temperature.^[^ ^130^ ^]^ The polymeric core can be fabricated using various techniques, including high‐pressure homogenization, nanoprecipitation, or solvent‐emulsion evaporation.^[^ ^131^ ^]^ A subsequent purification step is often necessary to remove unincorporated materials and isolate the final HNP product.^[^ ^132^ ^]^ In contrast, the one‐step method addresses several limitations of the two‐step approach, particularly in terms of scalability and process efficiency.^[^ ^133^ ^]^ The most prevalent one‐step techniques are solvent‐emulsion evaporation and nanoprecipitation. Among these, nanoprecipitation offers distinct advantages, including: Compatibility with automated microfluidic platforms; Production of nanoparticles with narrow size distributions; Utilization of non‐toxic solvents (e.g., ethanol); Enhanced encapsulation efficiency for macromolecules (plasmid DNA, mRNA, proteins). The nanoprecipitation process involves several critical steps: First, an aqueous phase containing dissolved lipid/PEG‐lipid components is heated above the lipid's gel‐to‐liquid transition temperature to ensure molecular homogeneity.^[^ ^134^ ^]^ This heated aqueous phase is then combined with an organic phase, triggering spontaneous self‐assembly of the polymeric core with subsequent lipid shell deposition. The resulting structure orients hydrophilic head groups outward and hydrophobic tails inward, creating a stable interface.^[^ ^135^ ^]^ Recent advancements in HNP fabrication have introduced more sophisticated techniques, such as microfluidic reactor technology, which enables the production of smaller, monodisperse particles with superior size control.^[^ ^136^ ^]^ These innovations continue to expand the potential applications and performance characteristics of hybrid nanoparticle systems. Microfluidic technology significantly improves the uniformity and encapsulation efficiency of LNPs through precise control of flow rates, chip design, and mixing parameters. A 2024 study showed that adjusting the total microfluidic flow rate (0.3–3.6 mL min^−1^) effectively improved the particle size (from 300 to 70 nm) and gene editing efficiency of Cas9 mRNA‐LNPs, with the highest editing efficiency at a total flow rate of 2.4 mL min^−1^.^[^ ^137^ ^]^

### 3.5 Lipid‐Based Nanoemulsions

Lipid‐based nanoemulsions (LBNEs) have emerged as a promising platform for drug delivery, particularly for enhancing the therapeutic potential of poorly water‐soluble drugs.^[^ ^138^ ^]^ These nanocarriers, typically ranging from 20 to 200 nm in diameter, consist of an oil phase uniformly dispersed and stabilized within an aqueous continuous phase through the action of surfactants or surfactant mixtures. The unique structural characteristics of these systems confer several advantages, including enhanced drug stability, potential for surface modification, and versatile functionalization capabilities.^[^ ^139^ ^]^ The nanoscale dimensions of these emulsions facilitate passive tumor targeting through the enhanced permeability and retention (EPR) effect, enabling selective accumulation of chemotherapeutic agents within the tumor microenvironment while minimizing systemic toxicity.^[^ ^140^, ^141^ ^]^ Furthermore, the surface of lipid nanoemulsions can be engineered with various targeting ligands to achieve active targeting, allowing for precise recognition of specific cancer cell markers and potentially enhancing therapeutic efficacy and selectivity.^[^ ^142^ ^]^ The inherent properties of lipid nanoemulsions, including their high kinetic energy resulting from their small size and large surface area, contribute to their effectiveness as drug carriers. Extensive preclinical investigations have demonstrated the therapeutic potential of these systems across various cancer types, including ovarian, lung, renal, and breast cancers.^[^ ^143^, ^144^, ^145^, ^146^ ^]^ The key advantages of lipid‐based nanoemulsions as drug delivery systems include: 1) Enhanced drug solubility and bioavailability; 2) Reduced systemic toxicity profiles; 3) Controlled and targeted drug release kinetics; 4) Potential to overcome multidrug resistance mechanisms due to their large surface area, superficial charge, elevated half‐life of circulation, specific targeting, and the imaging capacity of the formulation. For example, the co‐encapsulation of paclitaxel and baicalein in nanoemulsions can overcome multidrug resistance by augmenting oxidative stress and inhibiting P‐glycoprotein.^[^ ^147^ ^]^ Due to the vascularized nature of tissues surrounding cancer cells, nanoemulsions can readily gather in these tissues, using their size to pass through barriers. Nanoemulsions are capable of encapsulating anti‐angiogenic drugs within their core, which decreases toxicity and improves delivery of the payload.^[^ ^148^ ^]^ These characteristics collectively position lipid‐based nanoemulsions as a versatile and effective platform for advanced drug delivery applications in cancer therapy. Preclinical studies have shown that LBNEs loaded with capabatacel maintained an effective blood drug concentration for more than 72 h in a PDX model of renal cell carcinoma, and its hepatorenal toxicity was reduced by 40% compared with free drugs. LBNEs loaded with mRNA vaccines and PD‐L1 inhibitors induced tumor‐specific T cell responses in renal cell carcinoma models and overcame immune escape.^[^ ^149^ ^]^ Furthermore, LBNEs co‐loaded with sorafenib and the hypoxia‐activating prodrug T‐302 can synergistically sensitize the hypoxic tumor microenvironment and reverse drug resistance.^[^ ^150^ ^]^

### 3.6 Lipid Micelles

Lipid micelles represent self‐assembled nanostructures characterized by their amphiphilic nature, typically comprising a hydrophobic core enveloped by a hydrophilic shell.^[^ ^151^ ^]^ These micellar systems have garnered significant attention as versatile carriers for the delivery of bioactive molecules. Particularly noteworthy is their application in enhancing the solubility and bioavailability of poorly water‐soluble drugs, including various anticancer agents, thereby improving their pharmacokinetic profiles through both oral and parenteral administration routes.^[^ ^152^ ^]^ The inherent properties of lipid micelles enable effective passive targeting of tumor tissues, leveraging the characteristic features of tumor vasculature and the enhanced permeability and retention (EPR) effect. This targeting mechanism facilitates increased drug accumulation at the tumor site while simultaneously enhancing specificity and minimizing systemic toxicity. The targeting efficiency can be further augmented through surface modification of lipid‐core micelles with specific ligands, including antibodies, small molecules, and peptides, which enable active targeting of specific tumor cells.^[^ ^153^ ^]^ A particularly advantageous feature of certain lipid micelles is the incorporation of positively charged lipids that facilitate endosomal escape, allowing direct cytoplasmic delivery of encapsulated therapeutic agents. This characteristic is especially beneficial for intracellularly acting anticancer drugs, enhancing their therapeutic efficacy.^[^ ^154^ ^]^ In the treatment of renal cancer, the clinical application of hydrophobic drugs such as paclitaxel and cabazitaxel is often limited by their low solubility and poor bioavailability. The hydrophobic core of lipid micelles can efficiently encapsulate such drugs. For example, paclitaxel micelles have been shown to increase the tumor accumulation by 2 times in a mouse renal cancer model compared with traditional preparations, without increasing the toxicity to renal tubules.^[^ ^155^ ^]^ Sunitinib micelles have overcome the bottleneck of drug solubility through solubilization technology. Preclinical studies have shown that their half‐life is prolonged by 3 times and the blood drug concentration is more stable.^[^ ^156^ ^]^ The combination of several advantageous features—including straightforward preparation methods, active targeting capabilities, and enhanced stability—positions lipid‐core micelles as one of the most promising and effective drug delivery systems for cancer treatment.^[^ ^153^ ^]^ Their structural flexibility and functional versatility continue to make them a subject of intense research in the field of nanomedicine.

## 4 Targeted Delivery Mechanisms for LNPs on RCC

The treatment of renal cancer requires innovative strategies due to its aggressive and malignant characteristics. The traditional approaches offer non‐specific distribution of chemotherapeutic agents, which leads to both suboptimal therapeutic efficacy and significant side effects. Nanotechnology has provided researchers with promising avenues for enhancing the selectivity and efficacy of anticancer drugs. The delivery of therapeutic agents directly to the cancer cells can be achieved by passive and active targeting of drug‐loaded nanoparticles. These targeting techniques aim to maximize the concentration of drugs at the tumor site and improve the overall therapeutic index.^[^ ^157^ ^]^

### 4.1 Passive Targeting with Enhanced Permeation and Retention Effect

**Figure 6:**
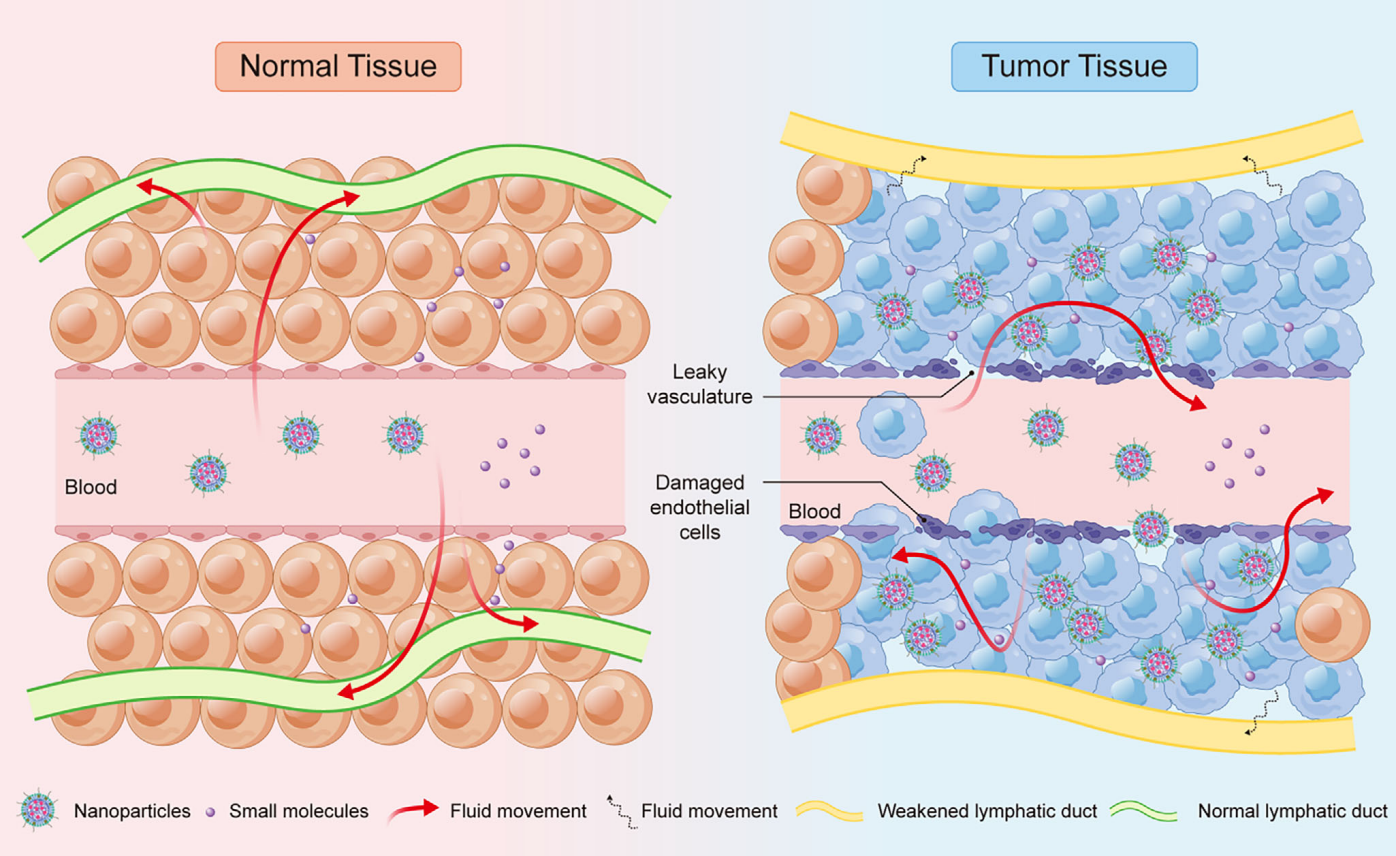
Passive targeting of a nano drug delivery system by enhanced permeation and retention effect.

The localization of the drug in passive targeting utilizes the inherent physiological characteristics of the tumor microenvironment. This includes techniques of designing nanoparticles of specific size, shape, charge, and surface properties, which can accumulate the drug at the tumor site. The nanoparticles preferentially accumulate at the tumor site due to the poor lymphatic drainage and leaky blood vessels in tumors, also known as the enhanced permeability and retention (EPR) effect. **Figure** **6** represents the EPR effects and the passive targeting of nanoscaled drug carriers. Nanoparticles between 10 and 200 nm can utilize the EPR effect and can be accumulated only in tumor tissues and not in healthy tissues. The passive accumulation of LNPs in the tumor microenvironment is further potentiated due to acidic pH and higher interstitial pressures. For example, doxorubicin‐loaded LNPs can accumulate in renal cancer in situ through an EPR effect, with concentrations 4 to 6 times higher than in normal kidney tissue.^[^ ^158^, ^159^ ^]^ Hence, the encapsulation of therapeutic molecules inside nanocarriers can prolong circulation time, enhance drug stability, and increase bioavailability.^[^ ^160^ ^]^ To leverage the highly vascularized nature of RCC tumors, LNPs should be engineered with optimized hydrodynamic sizes (30–100 nm) to penetrate the abnormal vasculature while minimizing renal clearance. Additionally, PEGylation enhances systemic circulation time and reduces macrophage‐mediated clearance, further improving tumor accumulation.

### 4.2 Active Targeting

Active targeting represents a sophisticated strategy in nanomedicine, involving the surface modification of nanoparticles with specific ligands such as small molecules, antibodies, or peptides. These engineered delivery systems demonstrate remarkable specificity by selectively binding to overexpressed receptors on cancer cell surfaces. The targeting efficiency is significantly enhanced through the conjugation of various ligands, including transferrin, folate, and specific peptides or proteins, to the nanoparticle surface.^[^ ^25^, ^161^ ^]^ This ligand‐receptor interaction facilitates enhanced cellular internalization of encapsulated therapeutic agents, thereby improving their therapeutic efficacy. The internalization process primarily occurs through receptor‐mediated endocytosis, with the specific uptake mechanism varying according to the cancer cell type and location.^[^ ^25^ ^]^ For instance, folate receptors exhibit significant overexpression in the proximal tubules of renal tumors.^[^ ^25^, ^161^, ^162^ ^]^ Consequently, folate‐modified nanoparticles demonstrate preferential accumulation in damaged kidney cells, with substantially higher concentrations observed in renal tissue compared to hepatic tissue. Similarly, nanoparticles functionalized with chitosan and glucosamine exhibit specific interactions with the megalin‐cubilin complex present in the epithelial cells of proximal tubules. This targeted interaction results in enhanced renal accumulation while minimizing hepatic uptake, demonstrating the precision of active targeting strategies.^[^ ^163^ ^]^ These examples illustrate the potential of ligand‐modified nanoparticles to achieve tissue‐specific drug delivery, highlighting their importance in developing targeted cancer therapies. In addition to enhancing tumor‐specific uptake, lipid nanoparticle‐based drug delivery offers a promising solution to overcome TKI resistance in RCC. The co‐encapsulation of TKIs with ABC transporter inhibitors, such as Tariquidar, can effectively counteract drug efflux mechanisms.^[^ ^164^ ^]^ Moreover, pH‐sensitive lipid nanoparticles, designed to release therapeutic payloads in the acidic tumor microenvironment (pH 6.5–6.8), enhance site‐specific drug release, reducing systemic toxicity while improving treatment efficacy.^[^ ^158^ ^]^ Besides, recent studies have explored lipid nanoparticle‐mediated gene silencing approaches to enhance RCC immunotherapy. The delivery of small interfering RNA (siRNA) targeting PD‐L1 or MUC1 via lipid nanoparticles effectively reduces immune evasion, while STING agonist‐loaded formulations promote innate immune activation, fostering a robust antitumor response.^[^ ^165^, ^166^ ^]^ In addition, the total number of lncRNAs is increasing as well due to more sensitive detection methods and is now greater than the sum of all protein‐coding genes. lncRNAs are mainly transcribed by RNA polymerase II and may subsequently undergo post‐transcriptional modifications. They have been detected in the nucleus, nucleolus, cytoplasm, and mitochondria. There is rising evidence suggesting a mechanistic link between cancer and lncRNA dysregulations. This makes lncRNA molecules potential therapeutic targets and biomarkers.^[^ ^167^ ^]^

## 5 Controlled Drug Release Mechanism of LNPs for RCC Based on Latest Studies

In recent years, significant progress has been made in the study of the drug release mechanism of lipid nanoparticles (LNPs) in the treatment of kidney cancer, especially in the aspects of targeted delivery, microenvironment response release and drug resistance regulation. pH‐sensitive LNPs remain electrically neutral in neutral blood before entering the acidic microenvironment of RCC. Then lipid protonation leads to the destruction of membrane structure, triggering rapid drug release. It is reported that DOPE‐modified LNPs were designed to achieve sustained local release of the drug in a mouse model of kidney cancer for up to 3 weeks, with an 80% reduction in postoperative recurrence rates.^[^ ^159^ ^]^ In a groundbreaking study conducted in 2024, Qingfeng Fu and colleagues investigated the therapeutic potential of sunitinib, a first‐line tyrosine kinase inhibitor for metastatic RCC, through innovative liposomal delivery.^[^ ^168^ ^]^ The researchers developed Liposome@Sunitinib, a targeted formulation administered via intra‐arterial injection, which demonstrated superior tumor‐specific accumulation compared to conventional intravenous administration. This targeted approach resulted in enhanced therapeutic concentrations at the tumor site while significantly reducing systemic side effects. The study reported remarkable tumor growth inhibition and progression control, attributed to the increased retention and accumulation of sunitinib in cancerous tissues.^[^ ^169^ ^]^ In a parallel development, Kuo‐Ching Mei et al. engineered a sophisticated liposomal system for co‐delivery of mitoxantrone and indoximod, representing a novel chemo‐immunotherapeutic approach.^[^ ^170^ ^]^ The formulation incorporated mitoxantrone, an anthraquinone derivative, alongside indoximod, a prodrug conjugated via cholesterol that serves as both an immunogenic cell death inducer and indoleamine 2,3‐dioxygenase pathway inhibitor. This dual‐loaded liposomal system demonstrated enhanced immunogenic responses, evidenced by increased ecto‐CRT expression and HMGB1 release. The formulation showed superior tumor suppression in both breast cancer and RENCA models, with notable activation of cytotoxic T‐cells and natural killer cells, as indicated by elevated IFN‐γ, perforin, and granzyme B expression levels. Addressing the critical challenge of drug resistance, Yihui Pan and colleagues (2023) identified IGFL2‐AS1, a long noncoding RNA overexpressed in sunitinib‐resistant RCC cells.^[^ ^47^ ^]^ Their innovative approach involved developing chitosan‐coated solid lipid nanoparticles (∼247 nm) for the targeted delivery of ASO‐IGFL2‐AS1. These nanoparticles demonstrated exceptional transfection efficiency and tumor‐specific delivery in patient‐derived xenograft models, effectively reversing sunitinib resistance through modulation of the TP53INP2 pathway. In another significant advancement, Xianhu Zeng et al. (2024) developed sialic acid‐conjugated, PEGylated nano‐micelles for siRNA delivery.^[^ ^45^ ^]^ These <40 nm spherical micelles, composed of ibuprofen and polyethyleneimine, effectively targeted Mucin1 overexpression in the tumor microenvironment. The formulation demonstrated remarkable efficacy in restoring sunitinib sensitivity through cholesterol pathway modulation, significantly inhibiting 786‐O cell xenografts and tumor metastasis while minimizing associated toxicities. LNPs deliver chloroquine (an inhibitor of autophagy lysosomes) that blocks drug‐induced autophagic protection mechanisms. In the 2024 trial, chloroquine LNP combined with sunitinib reduced the number of autophagic vesicles in kidney cancer cells by 60% and increased the apoptosis rate by 40%.^[^ ^171^ ^]^ Complementing these developments, Zhiping Yu and colleagues engineered anti‐G250 functionalized lipid nanobubbles for targeted RCC therapy.^[^ ^172^ ^]^ These nanobubbles demonstrated specific affinity for G250‐expressing cells, offering dual functionality in both therapeutic delivery and ultrasound‐based tumor imaging, as confirmed through immunofluorescence evaluations. **Table** **1** provides a comprehensive overview of recent studies investigating lipid nanoparticles for RCC, encompassing prophylactic, diagnostic, and therapeutic applications. These collective advancements underscore the transformative potential of nanotechnology in revolutionizing RCC treatment paradigms.

**Table 1:** Latest studies of Lipid Nanoparticles for RCCs.

Sr. No.	Authors	Year	Drug or Gene loaded into LNPs	Type of lipid nanoparticle	Cell line name	In vitro models/cell studies	Animal models	Key findings and outcomes	Refer‐ence
1.	Rachamala et al.	2024	mTOR inhibitor everolimus (E) and the survivin inhibitor YM155 (Y).	Liposome	RCC cell lines	Everolimus and YM155‐loaded liposomes manifested significantly higher sensitization of RCC cells	The developed liposomes improve the survival of the immunocompetent syngeneic mouse model of RCC and xenograft models.	Everolimus and YM155‐loaded liposomes were found to be involved in the induction of mitotic catastrophe by managing the downregulation of multiple cell cycle events.	[173]
2.	Zucaro et al.	2023	Tolvaptan, a V2R antagonist	Transferrin‐conjugated liposomes	CAKI‐1 clear cell renal carcinoma (ccRCC) cell line	Reduced cell proliferation. Transferrin‐conjugated liposomes considerably reduced the expression of cyclin A2, which has been established as a cell proliferation biomarker	None	Transferrin‐conjugated liposomes caused cytotoxic activity on the ccRCC cell line CAKI‐1 in vitro. The developed liposomes exhibited to be more effective than the free drug in terms of minimizing cell proliferation.	[174]
3.	Nakamura et al.	2023	STING agonist	Lipid nanoparticles (STING‐LNPs)	RENCA lung metastasis from the renal cancer	Improved activation of natural killer cells by the developed STING LNPs Significant therapeutic effects in treating the RENCA lung metastasis	Intravenous injection of STING‐LNPs significantly reduced the RENCA tumor colonies.	Monotherapies manifested no anticancer effect. Whereas, the natural killer (NK) cells were activated by the STING‐LNPs were considered to be a promising mechanism of the anti‐tumor effect.	[175]
4.	Xuedan et al.	2021	Neoepitope peptide Nes2LR	Liposome	RENCA renal cell line	Multivalent immunization with a number of neoepitope candidates, including Nes2LR, has been found more effective at inducing neoepitope‐specific CD8+ T cell responses.	BALB/c mice of 5–6 week old females were used for the two models; prophylactic and therapeutic vaccine tumor models. Whereas the 6–8 week old female BALB/c mice were used for the orthotopic RENCALUC+ kidney tumor model.	The immunization with the Nes2LR neoepitope elicited potent antitumor immune responses against subcutaneous RENCA tumors in the prophylactic and therapeutic Tumor models: Nes2LR immunization is more effective at eliciting antitumor immune responses and reducing lung metastases when employing the intra‐venous injection of RENCA cells.	[176]
5.	Pal K et al.	2019	Everolimus and Vinorelbine	Liposome	786‐O and A498 cells	Dual drug‐loaded liposomes demonstrated enhanced efficacy in reducing cell viability while being investigated in the 786‐O and A498 cell lines.	Dual drug‐loaded liposomes have been evaluated against the RCC xenografts. In the 786‐O xenograft model, the developed liposomes manifested a reduction in the tumor volumes. Likewise, the A498 xenograft model also demonstrated similar effects	Targeted liposomal formulation loaded with everolimus and vinorelbine presented to be a promising approach for the treatment of metastatic RCC.	[177]
6.	Kim et al.	2018	Novel TLR 7/8 bi‐specific agonists.	Nanoparticles	RENCA‐GL cells	MB49 renal cancer cell line, on which there has been no specific tumor antigens have been identified	MB49 cell lysates in combination with 522NPs were used to immunize the Balb/C mice, The 522NP+cell lysate immunization was found to be more effective in delaying tumor growth.	The therapeutic effectiveness of the Ad5mTRAIL‐mediated vaccine employing 522NPs manifested the efficacy of TLR7/8 agonist‐loaded NPs against the metastatic RCC.	[178]
7.	Yamada et al.	2017	Doxorubicin (DOX)	Liposome (MITO‐Porter)	OS‐RC‐2 renal cancer cell line, which is resistant to doxorubicin (DOX)	Mitochondrial delivery of DOX led to a significant reduction in cell viability resulting in the suppression of mitochondrial functions, a decrease in the production of ATPs, leading to the cytotoxic effects.	None	None	[179]
8.	Kulkarni	2016	Cabozantinib (multi‐receptor tyrosine kinase inhibitor XL184)	Liposome	769‐P, ACHN, A498 and Caki‐1	Loaded liposomes were found to be involved in the enhanced cytotoxic effects as compared to the XL184, leading to the sustained inhibition of phosphorylation of the Met, AKT, and MAPK pathways in RCC.	Renal tumor xenograft model	Liposomes induced prolonged inhibition of the tumor growth. This outcome is consistent with increased inhibition of kinase signalling pathways. Higher accumulation of liposomes in the tumor with lower toxicities.	[180]
9.	Hiromoto et al.	2015	Sorafenib and the Akt1 inhibitor A‐674563	Liposome (Lipofectamine 2000)	Human RCC ACHN cell line	ACHN/sh‐Akt1 cells manifested significantly higher sensitivity to sorafenib. The sorafenib treatment induced significant downregulation of antiapoptotic proteins (Bcl‐2, Bcl‐xL, and c‐Myc) in ACHN/sh‐Akt1 cells.	The xenograft model using human RCC ACHN cells	Treatment with sorafenib demonstrated significantly increased growth inhibition of the ACHN/sh‐Akt1 tumor. The apoptotic index in ACHN/sh‐Akt1 tumor was found to be significantly increased than ACHN/C tumor when treated with sorafenib liposomes.	[181]
10.	Akita et al.	2015	Plasmid DNA (pdna) encoding the soluble form of VEGFR (sflt‐1).	Liposomal nanoparticles	OS‐RC‐2.	A slight increase in the gene expression in the tumor was observed while replacing the hydrophobic scaffold of the ssPalm from myristic acid (ssPalmM) to α‐tocopherol succinate (ssPalmE). The LNP(ssPalmE) formulation was effective in suppressing tumor growth when delivering the CpG‐free pDNA encoding sFlt‐1.	Mice bearing tumors established from the RCC cell line OS‐RC‐2.	Optimizing the PEG modification on the LNP surface improved stability in blood circulation and achieved tumor‐selective gene expression. Tumor growth was significantly suppressed when the CpG‐free plasmid DNA (pDNA) encoding the soluble form of VEGFR (sFlt‐1) was delivered using the LNP formed with ssPalmE (LNP(ssPalmE)).	[182]
11.	Li et al.	2018	Akt1 antisense oligonucleotide (RX‐0201)	Folate receptor‐targeted lipid‐albumin nanoparticles (F‐LAN).	Folate receptor‐expressing KB cells	Cellular uptake and Akt1 inhibition of F‐LAN‐RX‐0201 were found to be greater than those of non‐targeted LAN‐RX‐0201, and F‐LAN‐RX‐0201 inhibited cell growth with an IC50 of 11.9 µm, while LAN‐RX‐0201 showed lower cytotoxicity with an IC50 of 32.0 µm	KB xenograft tumor model in mice. F‐LAN‐RX‐0201 exhibited increased tumor inhibition in comparison to LAN‐RX‐0201 at 16 mg kg^−1^, and F‐LAN‐RX‐0201 at 16 mg kg^−1^ showed comparable tumor inhibition compared to free RX‐0201 at a much higher dose of 90 mg kg^−1^.	The F‐LAN‐RX‐0201 exhibited promise as a therapeutic agent for tumor cells with greater folate‐receptor expression, with improved cellular uptake, Akt1 inhibition, and in vivo tumor inhibition.	[183]
12.	Abshire et al.	2017	Tyrosine kinase inhibitor (TKI) Sorafenib	Hermo‐sensitive liposomes	Human metastatic RCC cell line (786‐0)	The uptake of rhodamine B‐loaded liposomes by renal cancer cells was visualized using confocal and fluorescent imaging. The combination of TKI/TSLs and focused ultrasound (FUS) led to the least cell viability.	None	Evaluation of the efficacy of TKI/TSLs triggered by focused ultrasound in an in vitro tumor model of RCC. The combination of TKI/TSLs and FUS showed the highest cytotoxicity compared to other treatment groups.	[184]
13.	Soliman et al.	2020	Dual drug conjugate (DDC) of gemcitabine (GT) and 5‐fluorouracil (5FU).	Lipid hybrid nanoparticles	RCC cells (SNU‐349)	Dual drug conjugate‐loaded NPs showed superior anticancer activity against the RCC cell line, compared to the pure Gemcitabine and 5 5‐Fluorouracil or the physical mixture.	Zebrafish embryos	DDC‐loaded NPs exerted a potent apoptotic response in the zebrafish embryo model, compared to the pure drugs.	[185]
14.	Zhong et al.	2023	Indocyanine green (ICG)	Lipid nanobubbles	RCC 786‐O cells (CA IX‐positive) and ACHN cells (CA IX‐negative)	ACP/ICG‐NBs had specific binding activity and ideal affinity to the CA IX‐positive RCC cells (786‐O), but not to the CA IX‐negative RCC cells (ACHN).	786‐O tumor xenograft in mice. ACP/ICG‐NBs showed specific enhanced ultrasound and photoacoustic imaging effects in the 786‐O xenograft tumors.	CG‐loaded and anti‐CA IX peptide‐functionalized nanobubbles (ACP/ICG‐NBs) have multimodal ultrasound, photoacoustic, and fluorescence imaging capabilities, and can specifically enhance the ultrasound and photoacoustic imaging of RCC xenograft tumors	[186]
15.	Liu et al.	2014	Sorafenib	DPPC liposomes and HMC‐coated DPPC liposomes	RCC 786‐0, a human metastatic clear cell histology RCC cell line.	Sorafenib‐loaded PLGA particles and HMC‐coated liposomes exhibited significantly increased cell death. Sorafenib‐loaded PLGA and HMC‐coated liposomes killed 88.3% and 98% of the tumor cells, respectively.	None	Liposomes provide improved delivery and enhanced drug efficacy, especially with HMC coating. This improves cellular uptake by increasing the stability of particles and the in vivo circulation time.	[187]

## 6 Conclusion and Prospectives

This review highlights the remarkable progress in LNP technologies, including liposomes, SLNs, NLCs, HNPs, and lipid micelles, which have demonstrated significant potential in overcoming the limitations of traditional RCC therapies. A critical breakthrough lies in the ability of LNPs to reverse drug resistance—a hallmark of RCC progression. Strategies such as co‐delivery of chemotherapeutic agents with RNA therapeutics or immunomodulators (e.g., STING agonists) have demonstrated synergistic effects in restoring drug sensitivity and activating antitumor immunity. LNPs have revolutionized RCC treatment by leveraging their unique physicochemical properties, such as biocompatibility, tunable surface modifications, and controlled drug release kinetics. For instance, liposomal formulations like Liposome@Sunitinib and dual‐drug‐loaded systems (e.g., mitoxantrone‐indoximod liposomes) have shown enhanced tumor accumulation and immunogenic responses, significantly improving survival outcomes in preclinical models. Similarly, hybrid nanoparticles combining polymeric cores with lipid shells exemplify the synergy of material science and nanotechnology, enabling precise targeting of overexpressed receptors (e.g., folate, transferrin) and intracellular delivery of macromolecular therapeutics. The integration of stimuli‐responsive components (e.g., pH‐sensitive lipids, thermosensitive polymers) further refines spatiotemporal drug release, minimizing off‐target effects while maximizing therapeutic index.

At present, the lack of unified preparation standards and quality control systems leads to uneven quality of lipid nanoparticle products prepared by different laboratories and enterprises, making it difficult to achieve standardization and normalization in large‐scale production and clinical application. Scalability and reproducibility of manufacturing processes (e.g., microfluidic techniques, high‐pressure homogenization) require optimization to meet Good Manufacturing Practice (GMP) standards. Long‐term stability studies are needed to address issues such as lipid oxidation, drug leakage, and batch‐to‐batch variability. Furthermore, comprehensive toxicological assessments must evaluate the biodistribution, immune compatibility, and off‐target effects of functionalized LNPs, particularly those incorporating cationic lipids or synthetic polymers. Regulatory frameworks for nanomedicines also necessitate refinement to accelerate approval pathways while ensuring patient safety. Additionally, in clinical applications, its safety and toxicity issues still cannot be ignored. The components of lipid nanoparticles, such as lipids and surfactants, may trigger an immune response in the body, leading to adverse reactions such as fever and allergies.^[^ ^188^ ^]^ In addition, the metabolic pathways and long‐term safety of lipid nanoparticles in vivo remain unclear. For instance, its accumulation in organs such as the liver and spleen may have potential impacts on the functions of these organs. Whether long‐term use will trigger chronic toxic reactions also requires further research. For patients with renal cancer, as their kidney function has already been impaired, lipid nanoparticles may increase the burden on the kidneys and raise the risk of treatment.

To realize the full potential of LNPs in RCC management, concerted efforts are needed to: 1) Establish robust preclinical models (e.g., patient‐derived xenografts, 3D tumor spheroids) that recapitulate human disease complexity. 2) Conduct large‐scale clinical trials to validate efficacy and safety across RCC subtypes. 3) Foster interdisciplinary collaborations among chemists, biologists, clinicians, engineers, and regulatory experts to streamline translational workflows. For example, chitosan‐coated SLNs delivering antisense oligonucleotides (ASOs) effectively silenced resistance‐associated long noncoding RNAs, while sialic acid‐conjugated nanomicelles modulated cholesterol metabolism to resensitize tumors to tyrosine kinase inhibitors. These advancements underscore the potential of LNPs to disrupt resistance pathways at the genetic, metabolic, and immunological levels. The preparation process of lipid nanoparticles is complex, involving multiple raw materials and preparation methods, such as the thin‐film dispersion method, the emulsification evaporation method, microfluidic technology, etc. Different preparation processes can lead to significant differences in key quality parameters such as particle size, morphology, surface charge, and encapsulation efficiency of lipid nanoparticles, thereby affecting their in vivo pharmacokinetic and pharmacodynamic properties.^[^ ^189^ ^]^ All of these results require a multifaceted effort.

In conclusion, lipid‐based nanoparticles represent a shift in RCC therapeutics, offering unprecedented opportunities to overcome drug resistance, enhance targeting specificity, and improve patient outcomes. While challenges remain, the convergence of nanotechnology, molecular biology, and precision medicine holds immense promise for transforming RCC from a life‐threatening malignancy into a manageable chronic condition. Future research must prioritize translational innovation to bridge the gap between laboratory breakthroughs and clinical reality, ultimately ushering in a new era of personalized nanomedicine for renal cancer.

## Conflict of Interest

The authors declare no conflict of interest.

## Author Contributions

W.Y., Y.L. and Y.W. contributed equally to this work as co‐first authors. W.Y. wrote the original draft. Y.L. and Y.W. wrote, reviewed & edited the final manuscript. D.Z. investigate the project. Q.X. performed formal analysis. Y.W. supervised the project, did project administration and wrote, reviewed & edited the final manuscript. J.Z. and L.Z. wrote, reviewed & edited the final manuscript.
